# Revisiting miRNA Association with Melanoma Recurrence and Metastasis from a Machine Learning Point of View

**DOI:** 10.3390/ijms23031299

**Published:** 2022-01-24

**Authors:** Aigli Korfiati, Katerina Grafanaki, George C. Kyriakopoulos, Ilias Skeparnias, Sophia Georgiou, George Sakellaropoulos, Constantinos Stathopoulos

**Affiliations:** 1 Department of Medical Physics, School of Medicine, University of Patras, 26504 Patras, Greece; ceid3951@upnet.gr (A.K.); gsak@med.upatras.gr (G.S.); 2 Department of Dermatology, School of Medicine, University of Patras, 26504 Patras, Greece; georgiou@med.upatras.gr; 3 Department of Biochemistry, School of Medicine, University of Patras, 26504 Patras, Greece; g.kyriakopoulos@upnet.gr; 4 Laboratory of Molecular Biology, National Institute of Diabetes and Digestive and Kidney Diseases, Bethesda, MD 20892, USA; ilias.skeparnias2@nih.gov

**Keywords:** miRNAs, gene targets, cutaneous melanoma, artificial intelligence, metastasis, recurrence, NGS analysis, precision medicine

## Abstract

The diagnostic and prognostic value of miRNAs in cutaneous melanoma (CM) has been broadly studied and supported by advanced bioinformatics tools. From early studies using miRNA arrays with several limitations, to the recent NGS-derived miRNA expression profiles, an accurate diagnostic panel of a comprehensive pre-specified set of miRNAs that could aid timely identification of specific cancer stages is still elusive, mainly because of the heterogeneity of the approaches and the samples. Herein, we summarize the existing studies that report several miRNAs as important diagnostic and prognostic biomarkers in CM. Using publicly available NGS data, we analyzed the correlation of specific miRNA expression profiles with the expression signatures of known gene targets. Combining network analytics with machine learning, we developed specific non-linear classification models that could successfully predict CM recurrence and metastasis, based on two newly identified miRNA signatures. Subsequent unbiased analyses and independent test sets (i.e., a dataset not used for training, as a validation cohort) using our prediction models resulted in 73.85% and 82.09% accuracy in predicting CM recurrence and metastasis, respectively. Overall, our approach combines detailed analysis of miRNA profiles with heuristic optimization and machine learning, which facilitates dimensionality reduction and optimization of the prediction models. Our approach provides an improved prediction strategy that could serve as an auxiliary tool towards precision treatment.

## 1. Introduction

Cutaneous malignant melanoma (CM) is the most dangerous, heterogeneous and with a strong propensity to metastasize, skin cancer. Until today, the American Joint Committee on Cancer (AJCC) staging is the dominant synthetical index to CM prognosis and has been recently revised to its eighth edition, which improves staging, prognosis, and risk stratification [1]. Notably, staging is not the only prognosis determinant of CM, since survival outcomes and response to treatment can vary among patients even within the same stage due to its heterogeneity [2]. CM accounts for more than 75% of skin cancer deaths overall, with a 5-year survival rate of 23% in patients with late stage of the disease [3]. When detected at an early stage (stages I and II), prognosis is favorable; however, the survival rates for CM patients at stage III are rather ambiguous and at stage IV (metastatic) are poor. This heterogeneity is driven by environmental, genomic, epigenomic and transcriptomic factors and early CM detection is considered crucial for the reduction of the mortality rates [4]. Over the past two decades, our knowledge on CM has improved substantially. However, despite the intensive efforts to enhance targeted and immune therapy efficacy and the discovery of putative predictive biomarkers, the mechanisms of resistance and sensitivity remain poorly understood [5].

Precision medicine approaches exploring-omics data have emerged to identify diagnostic and prognostic biomarkers, which can lead to early disease detection, the better understanding of the underlying biological mechanisms and the application of individualized treatment protocols to patients with CM. Towards this goal, several studies have provided information using or combining genomics, transcriptomics and epigenomics analyses [6,7,8]. Based on the genomic context, four subtypes of CM have been identified: mutant BRAF, mutant RAS, mutant NF1 and triple WT (wild-type) based on the negativity of these mutant genes [6]. The transcriptomics analyses on the other hand support the existence of a non-overlapping classification consisting of four major subclasses: high-immune, normal-like, melanocyte inducing transcription factor (MITF)-high pigmentation and MITF-low proliferative [7]. The high-immune subtype has elevated expression of immune genes, the normal-like of genes expressed in surrounding normal cells, the MITF-high pigmentation subtype expresses cell-cycle genes and the MITF-low proliferative has high expression of cell-cycle and melanocyte differentiation genes. Finally, the aberrant methylation pattern of CpG islands of genes was investigated, along with histone modifications and expression levels of mRNAs and non-coding RNAs to demonstrate that 17 mRNAs and the miRNA hsa-mir-205 can distinguish primary and metastatic tumors better than methylation features [8]. Recently, a melanoblast specific signature of 43 genes was described to contribute to metastatic competence and to predict survival, in genetically engineered mouse models [9].

Through the advancement of sequencing methodologies, noncoding RNAs (ncRNAs) have emerged as among the most promising biomarkers for monitoring CM progression and recurrence, with miRNAs being the most well-studied class. Since their discovery, specific miRNAs have been recognized as dependable markers that help monitor cancer progression or regression. In CM, several different and highly conserved miRNAs exist which have been correlated with essential developmental processes and serve as diagnostic or prognostic markers, either alone or in combination with other types of biomarkers, based on their ability to target at the same time more than one mRNAs, as means of post-transcriptional gene expression regulation [10]. The enrichment of public databases like TCGA (The Cancer Genome Atlas) hosting sequencing data on CM (TCGA-SKCM project) provides a platform which can be exploited not only to address the role of specific miRNA expression patterns in correlation to different cancer stages or response to current therapies, but also to provide the basis for development and improvement of elaborate bioinformatics diagnostic tools [6]. However, tools that are based on only one specific class of biomarkers or only one-omics methodology have limitations and do not adequately support the diagnosis or prognosis of complex cancer types such as CM which, in turn, relies on a complex network of genes and regulatory ncRNAs. Therefore, multi-omics integrated analyses is a must for the identification of more accurate and specific CM prognostic biomarkers. Such approaches could include combinations of data on driver mutations, copy number variation, methylation patterns, and mRNA expression profiles, whereas integration of epigenomic and genomic data could identify prognostic CM—associated molecular subtypes [11,12]. Immune related aspects of CM have been also widely studied and have led to the generation of prognostic gene signatures consisting of 239, 25, 7 and 6 genes [13,14,15,16,17]. Moreover, a sex bias with improved survival of female CM patients has been observed, possibly due to specific mutations identified on X-linked epigenetic regulators [18,19]. Of note, studies on metastatic CM generated a metastasis-associated prognostic signature of 121 genes and moreover, mRNA, miRNA and methylation data extracted from TCGA were used as the basis to distinguish metastatic melanoma from primary tumors. Therefore, multi-dimensional analyses which examine multiple-omics profiles on the same samples are necessary to assess not only a better staging of CM but also to understand the persisting resistance to current therapies and recurrence of the disease [11].

Herein, we provide a comprehensive review on the role of specific miRNAs that contribute to the deregulation of gene expression in CM. In a next step, the value of specific miRNA signatures with possible role on recurrence and metastasis were examined in correlation to specific gene signatures that are currently known to play role in CM. After analysis of the TCGA-derived CM-related miRNASeq data with a statistical, bioinformatics and network analytics approach, we observed two distinct miRNA signatures, one strongly associated with metastasis and one strongly associated with recurrence. Incorporation of the TCGA mRNA transcriptomics profiles and analysis of the miRNAs’ putative targets gave us a better insight into the roles of the miRNAs of the two signatures. The resulted miRNA signatures were further analyzed with a multi-objective optimization ensemble classification method that was trained to predict CM recurrence and metastasis [20]. The trained classification models exhibited 5-fold cross validation accuracy of 91.51% and 97.39%, for predicting the recurrent and metastasis samples, respectively. Subsequent unbiased analysis on an external test set, using the trained classification models, revealed an accuracy of 73.85% and 82.09% in melanoma recurrence and metastasis prediction, respectively. Finally, the integration of TCGA clinical data in the miRNA signature of recurrent CM increased the predictive accuracy from 73.85% to 85.38%.

Collectively, the current analysis underlines the importance of rationalized integration of data from multiple sources which could provide novel and accurate means to prognosis of CM progression, recurrence and metastasis that could also apply for virtually any type of cancer. In addition, it highlights the importance of machine learning classification models for precision medicine approaches, and the opportunity to identify elusive, so far, important new biomarkers.

## 2. Datasets and miRNA Signatures from CM Patients

Several studies have established miRNAs as important diagnostic, prognostic and therapeutic markers in almost all known cancer types, including CM and other skin cancers [21]. The regulatory role of miRNAs includes modulation of gene expression in melanocytes, regarding specific immune response, apoptosis, cell cycle regulation and proliferation and cell invasion [22]. The oncogenic or tumor-suppressing role of specific miRNAs in the deregulation of melanocytes and the progression of CM is achieved through targeting of specific gene transcripts (mRNAs), which are involved in specific signaling pathways and cellular responses [23,24]. For example, several miRNAs which were found deregulated in CM can affect important signaling pathways that play role in resistance to known BRAF and MEK inhibitors [25]. In addition, subsets of deregulated miRNAs have been listed as potential biomarkers in response to treatments, as has become evident by the identification of circulating miRNAs to monitor tumor behavior [26,27,28,29,30]. Finally, microarray-based studies have suggested specific miRNA; however, these studies do not include recent miRNASeq data deriving from cohorts of patients with CM.

A thorough search in public repositories like Gene Expression Omnibus, TCGA and dbGaP for miRNASeq data deriving exclusively from CM biopsies resulted in 10 available datasets (Table 1). The TCGA-SKCM project includes various information (simple nucleotide variation, copy number variation, transcriptome profiling, biospecimen, sequencing reads, DNA methylation and clinical phenotypes) from 470 patients and miRNA-Seq data from 448 patients [6]. Additional GEO datasets deriving from studies on circulating miRNAs clearly demonstrated the utility of circulating cell-free microRNAs (cfmiRs) as potential blood biomarkers for stage III and IV CM patients and compared plasma of metastatic patients (some of whom with melanoma brain metastasis; MBM) before and during immune checkpoint blockade (ICB) therapy with normal healthy donor samples [31,32]. In addition, miRNA expression data from plasma and extracellular vesicles (GSE143231) were analyzed from patients with stage IV AJCC metastatic CM and compared to healthy donors [33]. In addition to the above few other studies that compared CM lymph node metastases with Merkel cell carcinoma (MCC; GSE53600) or FFPE samples to matched fresh frozen samples and a metastatic CM pair of matched samples (GSE45740) are also available [34,35]. Finally, two data sets compare primary CM biopsies with matched normal skin and common nevi (GSE36236; phs001550.v2.p1) [36,37].

Based on the accumulated sequencing data, recent efforts focused on the generation of miRNA signatures with the contribution of artificial intelligence (AI) tools to improve the prediction of metastasis, overall survival or recurrence of patients with CM and/or other cancer types. More specifically, an 11-miRNA signature was reported to discern primary melanoma from common nevi using formalin-fixed paraffin-embedded (FFPE) samples and microarray or miRNASeq experiments [38]. A similar study (GSE62370) resulted in 29 differentially expressed miRNAs (6 upregulated and 23 down-regulated) which provided a 4-miRNA signature with potential prognostic value [39]. Interestingly, an AI approach applied on miRNA ratios rather than the expression profiles of miRNASeq data from micro-dissected FFPE CM developed a model of 6 miRNAs that were either depleted or enriched. When combined with clinical features, this model achieved distinction of primary melanomas from common nevi with 81% sensitivity and 88% specificity [37]. Finally, analysis of the data available from TCGA provided 4 miRNAs that showed potential value to distinct metastatic from primary CM, whereas an attempt to specifically predict brain metastases resulted in a different 4-miRNA signature [40,41].

Predicting the overall CM patient survival is also of vital importance and can be clinically used for optimal treatment management. Several studies attempted to correlate specific miRNA expression profiles with the overall survival of CM patients, albeit with different outcomes (summarized in Table 2). Of note, among the miRNAs reported in the different signatures, only miR-155, miR-142-5p, miR-205, miR-203, miR-21-5p, miR-211, miR-125b, miR-150 and miR-16 are part of more than one signatures. Among those, miR-155 is known to inhibit proliferation, invasion, and migration of melanoma cells, while targets of miR-205 are involved in metastasis and survival of CM [42,43]. In addition, miR-142-5p is altered after UV radiation of the skin and miR-203 is significantly decreased in CM tissues, is associated with prognosis and has been shown to suppress metastasis in vivo [44,45,46]. Moreover, miR-21-5p is a well-studied oncomir reported to be overexpressed in several types of cancer and has been associated with promotion of cell cycle progression in CM [47]. Finally, miR-211 has been shown to contribute to BRAF inhibitor resistance in melanoma, while miR-125b and miR-150 act as tumor suppressors [48,49,50].

It is obvious that none of the existing reports can be used unambiguously as reference for clinical use, since they conclude on different miRNAs and this inconsistency can be attributed to a number of factors. Most of the studies rely on microarray experiments or even RT-qPCR for the discovery of a miRNA signature, thus profiling the expression of few prespecified miRNAs and possibly excluding from the signature other potential key miRNAs. In the same line, the selection of the profiled miRNAs is biased towards well-annotated miRNAs and miRNAs previously reported in the literature. More importantly, most studies rely solely on the differential expression analysis of the profiled miRNAs, which results to the generation of signatures with large numbers of miRNAs. To select key miRNAs among the ones differentially expressed, some studies select those with known role in CM, again excluding possibly discriminative miRNAs. Other studies select miRNAs by computing the correlation of each single miRNA to the target outcome, without considering their systemic and synergistic roles. To address these limitations, few studies employed machine learning tools for the discovery of signatures and the generation of classification models. However, traditional machine learning algorithms do not perform well with datasets from few samples and many miRNAs, which is a common case in miRNASeq data. The large number of miRNA profiles combined with the non-optimized choice of machine learning algorithm parameters can lead to overfitting and lack of generalization, i.e., the model learns to perfectly discriminate samples already provided for its training, but fails to classify new unknown samples. Therefore, external test data sets could be useful to assess the performance of machine learning models in a less biased way, an approach which is missing from most studies. In addition, most studies have been limited by the small number of samples either from specific miRNA datasets or from FF/FFPE samples. This limitation, combined with the differences in the analytical methodologies used, results in this variation and diversity of results and suggests that a more collaborative approach is required, using large datasets obtained from public databases such as TCGA.

## 3. Transcriptomics and Gene Signatures from CM Patients

The use of NGS for transcriptomics analysis and several elaborate bioinformatics tools have correlated the expression of several genes with CM progression and metastasis (presented in detail in Table 3). Specifically, two distinct gene signatures were generated using data from GEO datasets for the prognosis of CM, while the use of the TCGA dataset resulted in gene signatures to predict metastasis and overall survival [60,61,62,63]. Both datasets produced a 4-gene model (*ADAMDEC1*, *GNLY*, *HSPA13*, *TRIM29*) that is strongly correlated with survival prediction [64]. Similarly, co-expression analyses using publicly available RNA-seq data (GSE98394) and immuno-histochemistry from the Human Protein Atlas revealed 3 genes (*STK26*, *KCNT2*, *CASP12*) with potential value as predictors for accurate diagnosis and prognosis of CM [65]. Moreover, expression analysis of specific candidate genes from fresh frozen samples revealed a 9-gene signature (*KRT9*, *KBTBD10*, *DCD*, *ECRG2*, *PIP*, *SCGB1D2*, *SCGB2A2*, *COL6A6*, *HES6*), which could predict the clinical outcome [66]. Analysis of microarray expression data identified a signature of 28 genes (Table 3) for metastasis risk, while analysis of three chip datasets from the GEO database revealed 8 genes (*DSG3*, *DSC3*, *PKP1*, *EVPL*, *IVL*, *FLG*, *SPRR1A*, *SPRR1B*) with significant predictive value for metastatic transformation of CM [67,68]. A diverse signature of 6 genes (*ALDH1A1*, *HSP90AB1*, *KIT*, *KRT16*, *SPRR3*, *TMEM45B*) to predict metastasis was identified using publicly available transcriptomics data from biopsies of CM patients, using an AI approach and scanning a genome-wide protein-protein interaction network, followed by shortlisting of the most influential and most differentially expressed genes. The gene signature showed 87% classification accuracy and was further validated on an independent transcriptomic data set and highlighted the importance of AI tools for a more accurate prediction approach [69]. In addition, analysis of samples from the Leeds Melanoma Cohort (LMC) reported association of high *JUN* and *AXL* expression with poor prognosis and response to immunotherapy [70]. Finally, additional studies using either publicly available datasets or tissue samples, also reported gene signatures that could potentially predict clinical outcome or distinguish between metastatic and primary CM [71,72,73,74].

Most recently, studies using CM mouse models have led to the generation of similar gene signatures with potential use as prognostic tools for CM progression, recurrence, and metastasis. In more aggressive melanomas, a set of 4 upregulated genes (*IGF2BP1*, *PTMA*, *MYC*, *MITF*) was identified and a prognostic signature of 13 genes for metastatic CM was reported (*CDKN2A*, *CDKN2B*, *ZBTB16/PLZF*, *CDKN1A*, *TYR*, *ARNT2*, *MDM2*, *GPR143*, *RAB38*, *ANGPT2*, *MGAT5*, *POU4F1*, *SIX1*) [75,76]. In addition, the use of genetically engineered mouse models created a 43-gene signature that predicts patient survival and the clinical outcome of immune checkpoint inhibitors [9,77]. This set of genes is melanoblast-specific, and their expression may contribute to metastatic competence. More specifically, four different melanoma cell lines with different levels of sensitivity to immune checkpoint blockade (ICB) treatment were isolated and subsequent transcriptomics analysis revealed that the differentiation status of melanoma cells may determine the ICB benefit. Notably, the four mouse models were each found to correspond to a human CM phenotype (undifferentiated, neural crest-like, transitory, melanocytic) [77].

Genes reported in more than one signature include *SPRR1B*, *KCNT2*, *CRABP2*, *TRIM29*, *LTA4H* and *PDE3B*. Similarly with the miRNA signatures mentioned above, most research groups were limited to datasets of a few genes and followed different methodologies in an attempt to generate gene-specific signatures, leading to significant variations between gene clusters, suggesting that a more collective approach is needed.

## 4. AI-Based miRNA Signatures for Prediction of Melanoma Recurrence and Metastasis

To gain insight into the contribution of miRNAs reported so far in melanoma recurrence and metastasis, we analyzed data from the TCGA-SKCM project, which is the largest collection of integrated biological and clinical data from 470 melanoma patients. miRNA-Seq data are available for 448 patients [6]. For 4 of the patients, data from two biological samples have been deposited, resulting in a total of 452 samples. Information on tumor recurrence is available for 438 samples, among which 328 samples are from a recurrent tumor and 110 are not. Combined analysis of differential expression profiles and comparison of co-expression networks of miRNAs (see supplementary methods for details) revealed a 7-miRNA signature (miR-155, miR-205, miR-376b, miR-1226, miR-1306, miR-3652, miR-3917) associated with CM recurrence (Table 4). Differential expression analysis alone, even when a strict cutoff was used (adjusted p value 0.05), resulted in 203 deregulated miRNAs. Comparison of the two miRNA co-expression networks resulted in 31 miRNAs with altered co-expression patterns. The 7 signature miRNAs are those that are both statistically significant, differentially expressed in the two recurrence conditions and significantly altered when comparing the miRNA co-expression network of the recurrent samples with that of the non-recurrent samples (see Supplementary Materials for details).

Subsequent analysis led to the identification of characteristic miRNAs in tumor metastasis. Among the 452 samples, 353 originated from metastatic tumors and 97 from primary tumors. The same analysis steps (see Supplementary Materials for details) led to a signature of 8 miRNAs (miR-186, miR-671, miR-760, miR-944, miR-1976, miR-3610, miR-3615, miR-6842) associated with metastasis which, with few exceptions, have been previously reported with important biological roles in melanoma and other cancer types (Table 5). Our analysis showed that 200 miRNAs were deregulated in metastatic samples compared to primary tumors and 24 miRNAs had altered co-expression patterns when the miRNA co-expression network of metastatic samples was compared with that of primary tumor samples.

### 4.1. Specific miRNAs Expression Patterns Regulate Melanoma Related Genes

In a next step, the analysis was enriched by incorporating TCGA RNASeq data from the same samples to identify genes that negatively correlated with the miRNA patterns using the Spearman correlation method with Rho < −0.25. Subsequent target prediction analysis between the signature miRNAs and negatively correlated mRNAs resulted in two sets of putative miRNA gene targets (see Supplementary Materials for details). Functional analysis revealed statistically significant (*p*-value < 0.05) gene ontology enrichment in terms such as melanoma and melanogenesis. Furthermore, these genes are involved in several important biological processes and molecular functions, such as transcriptional regulation and signaling, a finding that provides the basis for further experimental validation.

**Figure 1:**
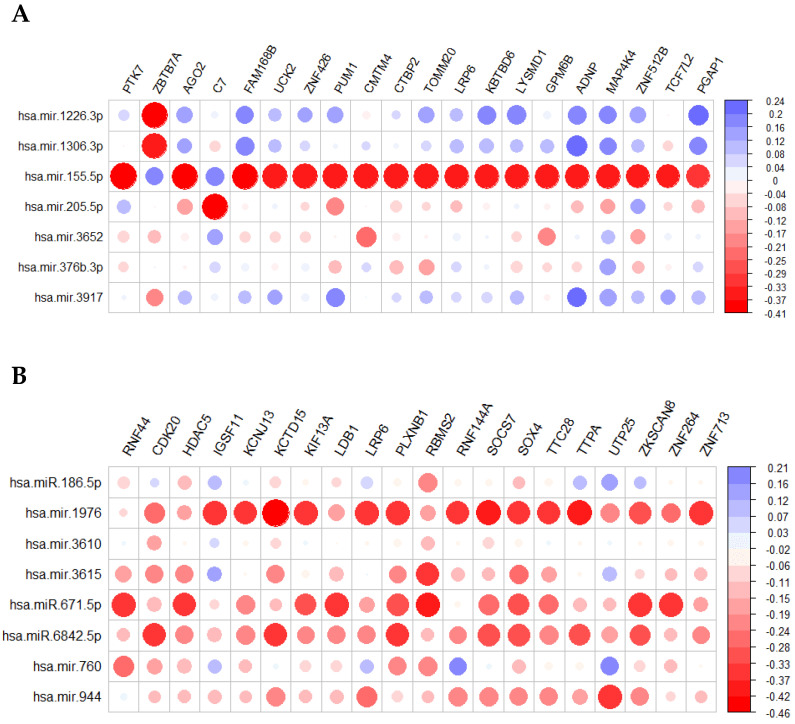
miRNAs of the recurrence (**A**) and the metastasis (**B**) signatures target genes with high negative correlation. The *y*-axis on the right represents the correlation coefficient Spearman Rho.

The putative target genes with the most negative correlation to recurrence were PTK7, ZBTB7A, AGO2, C7, FAM168B, UCK2, ZNF426, PUM1, CMTM4, CTBP2, TOMM20, LRP6, KBTBD6, LYSMD1, GPM6B, ADNP, MAP4K4, ZNF512B, TCF7L2, PGAP1 (Figure 1A, red circles). Similarly, the putative target genes with the most negative correlation to metastasis were RNF44, CDK20, HDAC5, IGSF11, KCNJ13, KCTD15, KIF13A, LDB1, LRP6, PLXNB1, RBMS2, RNF144A, SOCS7, SOX4, TTC28, TTPA, UTP25, ZKSCAN8, ZNF264, ZNF713 (Figure 1B, red circles). Genes which are common targets of the two miRNA signatures include ANKRD50, BPTF, CAMSAP2, CLCN3, CREBBP, FARP1, GAB2, IGF1R, IGSF11, LRP6, MAU2, MBTD1, NECTIN1, PIP4K2B, PRKCE, PUM1, RFTN2, SLC39A10, TEAD1, TRIM13, UCK2, YAP1, ZBTB7A, ZNF250, ZNF512B, ZNF609, ZNF618, ZNF704 which, after gene ontology analysis, are enriched in adherence junction, transcription initiation from RNA polymerase II promoter, signal transducer activity and hippo signaling.

**Figure 2:**
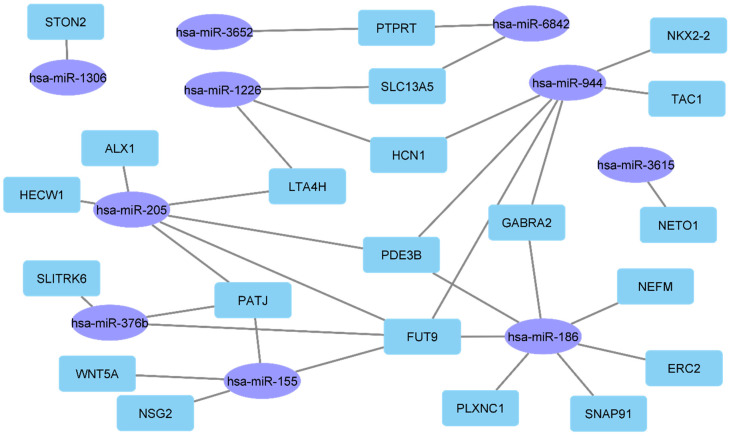
The recurrence (**left**) and metastasis (**right**) signature miRNAs target signature genes which in turn contribute to metastatic competence and can predict patient survival.

Interestingly, the analysis performed indicated several miRNAs that can target signature genes with a contribution to metastatic potential and could indicate patient survival. In particular, several genes, including *PATJ*, *FUT9*, *GABRA2*, *PDE3B* and *LTA4H* were among the putative targets of miR-155, miR-186, miR-205, miR-376b, miR-944, miR-1226, miR-1306, miR-3615, miR-3652 and miR-6842 (Figure 2). Most importantly, these genes are members of a previously reported gene signature, which is derived from four immunosuppressed melanoma mouse models and represents major molecular and phenotypic subtypes of human CM [77]. In addition, several of these genes such as FUT9 and PDE3B have been correlated to cell adhesion and angiogenesis, GABRA2 has been associated with the probability of survival in colorectal cancer and PATJ is a regulator of epithelial cell microtubule elongation and cell migration.

### 4.2. Contribution of AI in the Prediction of Melanoma Recurrence and Metastasis

Based on the preceding, a multi-objective ensemble optimization method was used and recurrence and metastasis prediction models were generated using the values of the signature miRNAs reads per million (RPM) as input (see Supplementary Materials for details) [20]. The applied method is based on the hybrid combination of heuristic optimization and nonlinear machine learning classification methods [97]. Specifically, it is an ensemble dimensionality reduction technique employing a heuristic optimization algorithm to (a) identify the optimal feature subset to be used as input to the classifiers, (b) to select the most appropriate classifier among support vector machines (SVM) and random forests and (c) to select the optimal parameters for the classifier, namely C and gamma of SVM and number of trees for random forests. This approach allows both an unbiased and an optimized selection of the classification method and its parameters. To handle the multiple objectives of maximization of predictive performance, minimization of selected features and simplicity of the classification model, we deployed a multi-objective Pareto-based approach to reveal all the non-dominated solutions of the above-stated optimization goals [20]. These solutions were then combined in an ensemble manner to predict tumor recurrence and metastasis. This ensemble approach allows combining more than one classification model to optimize prediction performance. For recurrence, the cross-validation accuracy achieved was 91.51% with 92.65% specificity and 91.29% sensitivity. Machine learning algorithms can learn linear and non-linear patterns from the data provided to them for their training. Thus, testing the performance of a classification model with the training data, or even with a cross-validation strategy favors the performance of the classifier. A common practice to evaluate a classifier more rigorously is to calculate the predictive performance on samples not previously seen by the algorithm. The respective metrics in the external test set (i.e., samples not seen before by the algorithm) were 73.85% accuracy with 79.09% specificity and 88.78% sensitivity. For metastasis, the cross-validation accuracy achieved was 97.39% with 96.67% specificity and 98.38% sensitivity. The respective metrics in the external test set were 88.78% accuracy with 82.40% specificity and 98.10% sensitivity (Table 6).

To test whether incorporating clinical characteristics into the analysis would improve the results of recurrence prediction, the inclusion of various demographic and staging related AJCC characteristics was also tested. The best prediction model chose to include the following features in the miRNA signature: sample type, AJCC T stage and AJCC stage. As a result, we observed increased predictive performance to 96.51% accuracy, 96.67% specificity and 98.38% sensitivity when using cross validation and 85.38% accuracy, 88.35% specificity and 92.86% sensitivity in the external test set (Table 6). Thus, providing the classifier with the relative expressions of the 7 signature miRNAs for a patient would predict with an accuracy of 73.85% whether or not it is a recurrent tumor, and additionally providing the classifier with the aforementioned three clinical features in addition would predict recurrence with an accuracy of 82.09%.

## 5. Discussion

We report, herein, the diagnostic and prognostic potential of miRNAs as reliable biomarkers in CM and discuss their functional correlation with previously reported gene expression signatures. Incorporation of such signatures in clinical practice could assist clinicians to take more informed decisions. However, different studies based on different data, or even based on the same data but using different bioinformatics tools result in inconsistency regarding specific signatures. Overcoming these limitations requires larger patient cohorts, multi-omics data instead of single-omics, more elaborate bioinformatics analysis and more sophisticated artificial intelligence tools.

With a special focus on CM recurrence and metastasis, we applied a comprehensive data analysis approach to publicly available NGS data, integrating statistical, bioinformatics and network analytics. We analyzed the largest available miRNA-Seq data cohort of melanoma patients and identified one miRNA signature of recurrence and one miRNA signature of metastasis. Integration of RNA-Seq data from samples from the same patients allowed functional enrichment of these signatures. The targets of miRNA signatures are related to melanoma, melanogenesis, transcriptional regulation and signal transduction. In addition, miRNAs of these signatures are associated with response to several promising immunotherapies. Of note, the identified miRNAs can potentially target signature genes which, in turn, are involved in metastasis and could be used to predict patient survival.

The emerged 7 miRNAs signature related to recurrence (miR-155, miR-205, miR-376b, miR-1226, miR-1306, miR-3652, miR-3917; Table 4) was previously reported to regulate important biological processes and to drive cancer progression. Interestingly, miR-1226 targets expression of the mucin 1 oncoprotein (MUC1) and induces cell death [82]. Recently, the potential mechanism of miR-1226-3p regulating MUC1 which, in turn, affects resting of dendritic cells, was shown to play an important role in soft tissue sarcoma (STS) recurrence [81,82]. On the other hand, miRNAs associated with prognosis and inhibition of carcinogenesis, such as miR-155 and miR-205 were also identified. Interestingly, both miRNAs have been part of a 6 membered cluster of serum miRNAs that can detect metastatic melanoma [98]. Stable inhibition of miR-155 has been correlated with retarded glucose metabolism and reduced tumor growth in vivo, while ectopic expression of miR-205 significantly inhibits cell proliferation and anchorage independent growth as well as cell invasion [78,79]. Finally, the signature that we propose to predict CM recurrence includes miR-376b, miR-1306 and miR-3917 which have been also proposed as biomarkers in several cancers and were found to be correlated with apoptosis and autophagy [80,83,84].

On the other hand, a group of 8 miRNAs constitutes the proposed signature for metastasis prediction (miR-186, miR-671, miR-760, miR-944, miR-1976, miR-3610, miR-3615, miR-6842; Table 5). Among those, miR-186, miR-671, miR944 and miR-3610 are the most well characterized. Of note, miR-186 regulates TGFβ by suppressing SMAD6-7 colorectal and inhibits cell proliferation in melanoma and in the same line, upregulation of miR-671 slows down proliferation and metastasis of A375 melanoma cells [81,85]. miR-3610 has been associated with sumoylation and it was recently also included in a molecular signature of head and neck cancer [95]. Finally, miR-944 has been reported to be deregulated exhibiting either tumor suppressive or oncogenic function in human malignancies [90,91,92].

Training classification models using these signatures allowed the prediction of melanoma recurrence and metastasis with high accuracy (accuracy 91.51% and 97.39%, respectively, with 5-fold cross validation). Testing our models in an external test set, i.e., completely unseen patients’ samples for the trained models has an accuracy of 73.85% and 82.09% in predicting melanoma recurrence and metastasis, respectively. In addition, the prediction models that we produced have the potential to stratify patients, by identifying those who are at risk for tumor recurrence and metastasis. The ability of the proposed hybrid dimensionality reduction and classification technique to identify the optimal subset of clinical features and combine them in an optimal and non-linear manner allowed the improvement of the predictions. Moreover, the integration of additional clinical data in the recurrence miRNA signature raised the predictive accuracy in the external test set from 73.85% to 85.38%. The combination of gene expression profiles with clinical data significantly improves the predictive performance as has been previously highlighted by similar studies on CM [99,100]. In our study, the observed improvement highlights the need for integration of data from multiple sources, such as multi-omics, clinical and imaging data, to provide a more comprehensive and accurate description of the biological processes underlying the disease and lead to more informative biomarkers.

Several recent studies support the value of circulating miRNAs as potential biomarkers of CM [28]. As such, the use of single cell data could contribute further to our understanding of the clonality and heterogeneity of CM and could explain molecular events underlying recurrence and metastasis [101]. The miRNA signatures reported herein include miRNAs that were previously shown to participate in melanoma occurrence and progression, as well as novel miRNAs. The strict filtering performed in the present study resulted in clusters of miRNAs that are significantly correlated with metastasis and recurrence and are strong predictors of these events, with an accuracy higher than 70%. All the above suggest that their involvement in melanoma progression is significant and could help not only the clinicians to better predict the prognosis of melanoma patients for more accurate therapeutic approaches, but also the researchers to better understand the complex gene networks which are deregulated in melanoma.

## 6. Conclusions

Recent efforts to perform transcriptomics profiling on large cohorts of CM patients enable the better understanding of melanoma biology. Bioinformatics, heuristic optimization, and machine learning classification models, when combined and applied to big data provide unique opportunity to identify potentially useful miRNA signatures as biomarkers from precision diagnosis and timely prognosis of metastasis or recurrence in response to treatment. Several studies evaluating the prognostic significance of miRNAs in CM recurrence and metastasis had inconsistent results, possibly attributed to methodological variations. Different studies based on different data, or even based on the same data, but with different analytics tools come up with different inconsistent signatures. Larger cohorts of patients, multi-omics instead of single-omics data, better bioinformatics tools and better AI tools are needed to overcome these limitations. However, even in this case, experimental low-throughput validation is required before such signatures can be incorporated in clinical practice.

## Figures and Tables

**Table 1:** Data origin and sample description of the available miRNA-Seq datasets.

miRNASeq Dataset	Melanoma Samples	Control Samples	Other Diseases Samples	Biomaterial
TCGA-SKCM [6]	448 melanoma patients	-	-	Tissue
GSE157370 [31]	47 stage III and IV melanoma patients (pre-treatment samples) and 111 CII post-treatment samples from the same patients	73 healthy donors	-	Plasma
GSE150956 [32]	36 + 24 pre-operative MBM patients’ plasma samples	48 Normal (cancer-free) donor plasma and serum plasma	49 other cancer types that had brain metastasis and glioblastomas	Plasma
	24 MBM tissues	-	-	Tissue
	20 pre-and post-treatment plasma and 14 urine samples collected from metastatic melanoma patients receiving CII	8 Normal (cancer-free) urine samples	-	plasma and urine
GSE143231 [33]	10 metastatic melanoma AJCC stage IV patients	five HDs	-	plasma and EVs
GSE53600 [34]	1 melanoma lymph node metastases	1 normal skin	6 MCC lymph node metastases, SCC and BCC primary cutaneous lesions	Frozen tissue
GSE45740 [35]	1 metastatic melanoma		7 breast invasive ductal carcinoma, renal clear cell carcinoma, lung adenocarcinoma, prostate adenocarcinoma and sarcoma of thigh	paired FFPE and fresh frozen samples
GSE36236 [36]	19 primary cutaneous melanomas biopsies/excisions	matched normal skin and common nevi	-	FFPE tissue
phs001550.v2.p1 [37]	8 melanomas	7 intact adjacent benign nevi	-	FFPE microdissected regions

**Table 2:** CM miRNA signatures as biomarkers of survival.

miRNA Signature	Significance	Datasets	Samples	Reference
miR-31-5p, miR-21-5p, miR-211-5p, miR-125a-5p, miR-125b-5p and miR-100-5p (miRNA ratios)	distinction of melanomas from nevi	FFPE phs001550.v2.p1 (miRNASeq)	41 nevi and 41 melanomas	[37]
miR-155-5p, miR-9-5p, miR-142-5p, miR-19a-3p, miR-134-5p, miR-301a-3p, miR-205-5p, miR-203a-3p, miR-27b-3p, miR-218-5p, and miR-23b-3p		FFPE (microarray)	5 cutaneous nevi and 27 primary melanomas	[38]
miR-142-5p, miR-550a, miR-1826, and miR-1201		GSE62370 (microarray)	9 congenital nevi and 92 primary melanomas	[39]
miR-205, miR-203, miR-200a-c, and miR-141	distinction of metastatic from primary melanomas	TCGA (miRNASeq)	97 primary and 350 metastatic melanomas	[40]
miR-150-5p, miR-15b-5p, miR-16-5p, and miR-374b-3p	prediction of brain metastases	IMCG GSE62372 (microarray)	256 primary melanomas	[41]
miR-125b, miR-200c and miR-205	prediction of overall survival	FF (RT-qPCR)	65 primary and 67 metastatic melanomas	[51]
miR-202, miR-206, miR-3681, miR-122 and miR-1246		TCGA (miRNASeq)	448 melanomas	[52]
miR-16, miR-211, miR-4487, miR-4706, miR-4731, miR-509-3p and miR-509-5p		FFPE (RT-qPCR)	86 melanomas	[53]
miR-497, miR-145, miR-342-5p, miR-150, miR-155 and miR-455-5p	prediction of post-recurrence survival	FFPE (microarray)	59 melanomas	[54]
miR-25, miR-204, miR-211, miR-510 and miR-513c	prognostic biomarker in cutaneous melanoma	GSE35579 (microarray)	11 benign nevi and 41 melanomas	[55]
miR-10b		FF (microarray)	20 non-metastasizing and 20 metastasizing primary melanomas	[56]
miR-338, let-7, miR-365, miR-191, miR-193b-3p and miR-193a-3p		FF, GSE19387 (microarray)	32 samples from regional lymph node metastases	[57]
miR-150-5p, miR-142-3p and miR-142-5p		FF (microarray)	84 samples from lymph node metastases	[58]
miR-21-5p, miR-424-5p and let-7b	associated with invasive and aggressive phenotype	FF, GSE36236 (miRNASeq)	12 normal skin, 13 common nevi, 17 dysplastic nevi, 45 melanomas in situ and 80 primary cutaneous melanomas	[59]

FFPE: Formalin-Fixed Paraffin-Embedded; FF: Fresh Frozen; IMCG: Interdisciplinary Melanoma Cooperative Group; RT-qPCR: reverse transcription quantitative Real time PCR.

**Table 3:** Gene signatures as putative prognostic biomarkers in CM.

Gene Signature	Significance	Datasets	Samples	Reference
*BAX*, *CALM1*, *CALM3*, *FN1*, *PRKCA*, *RB1*, *VEGFA*, *IGF1*	prognostic biomarker in cutaneous melanoma	GSE3189, GSE4570 and GSE4587	28 nevi and 58 melanoma samples	[60]
*CXCR4*, *IL7R*, *PIK3CG*		GSE65904	214 melanoma samples	[61]
*IGF2BP1*, *PTMA*, *MYC*, *MITF*	elevated levels in more aggressive phenotypes	mouse model		[75]
*KRT9*, *KBTBD10*, *DCD*, *ECRG2*, *PIP*, *SCGB1D2*, *SCGB2A2*, *COL6A6*, *HES6*	prediction of clinical outcome	FF	135 melanomas	[66]
*RHBDL3*, *GPR64*, *ANKRD30A*, *PRKCD*		TCGA, GSE22138, GSE54467, GSE65904 and E-MTAB-4725	102 melanomas + 565 samples (for confirmation)	[71]
*DSG3*, *DSC3*, *PKP1*, *EVPL*, *IVL*, *FLG*, *SPRR1A*, *SPRR1B*	distinction of metastatic from primary melanomas	GSE46517, GSE15605, GSE8401	109 primary and 136 metastatic skin melanomas	[68]
*ALDH1A1*, *HSP90AB1*, *KIT*, *KRT16*, *SPRR3*, *TMEM45B*		GSE15605, GSE7553, LMC and TCGA	20 normal samples, 867 primary and 419 metastatic melanomas	[69]
*ABCC3*, *CAPS2*, *CCR6*, *CDCA8*, *CLU*, *DPF1*, *PTK2B*, *SATB1*, *SYNE1*	prognostic biomarker in metastatic melanoma	TCGA, GSE19234, and GSE22153	556 cutaneous melanomas	[62]
*STK26*, *KCNT2*, *CASP12*		GSE98394	27 common required nevi and 51 primary melanomas	[65]
*BAP1b*, *MGP*, *SPP1*, *CXCL14*, *CLCA2*, *S100A8*, *BTG1*, *SAP130*, *ARG1*, *KRT6B*, *GJA1*, *ID2*, *EIF1B*, *S100A9*, *CRABP2*, *KRT14*, *ROBO1*, *RBM23*, *TACSTD2*, *DSC1*, *SPRR1B*, *TRIM29*, *AQP3*, *TYRP1*, *PPL*, *LTA4H*, *CST6*		FFPE	268 melanoma samples	[67]
*A2M*, *DUSP6*, *HLA-B*, *SERPINE2*, *SLC26A2*		GSE115978	31 melanoma samples	[72]
*CDKN2A*, *CDKN2B*, *ZBTB16/PLZF*, *CDKN1A*, *TYR*, *ARNT2*, *MDM2*, *GPR143*, *RAB38*, *ANGPT2*, *MGAT5*, *POU4F1*, *SIX1*		GSE149884	murine melanoma cell lines	[76]
*SERPINH1*, *HOXC10*, *MYH10*, *EPHB2*, *SRPX2*, *CGREF1*, *DDR2*, *P4HA2*, *IGSF10*, *OSM*, *ADORA3*, *RECK*, *KDELR3*, *TMEM8*, *SMARCA1*, *JAZF1*, *FKBP7*, *ZFP449*, *TRIQK*, *REN1*, *IGF2BP2*, *GRB10*, *DPYSL4*, *CMBL*, *PDE3B*, *DAB2*, *PPP1R9A*, *QPRT*, *PEG10*, *NID1*, *EFNB3*, *COLGALT2*, *DBN1*, *C1QTNF3*, *CDC7*, *MDK*, *GULP1*, *HOXD13*, *EYA4*, *DEPDC1A*, *CRABP2*, *ATP10B*, *TTYH1*, *SLITRK2*, *ELOVL2*, *STK32B*	prediction of overall survival	GSE140193, GSE25164	genetically engineered mouse model	[9]
*IL15*, *CCL8*, *CLIC2*, *SAMD9L*, *TLR2*, *HLA.DQB1*, *IGHV1-18*, *RARRES3*, *GBP4*, *APOBEC3G*		TCGA	470 melanomas	[63]
*ADAMDEC1*, *GNLY*, *HSPA13*, *TRIM29*		GSE7553, GSE46517, and GSE15605	17 normal skin and 202 melanomas	[64]
*IQCE*, *RFX6*, *GPAA1*, *BAHCC1*, *CLEC2B*, *AGAP2*		TCGA, GSE19234 and GES65094	485 melanomas	[73]
*CCR9*, *CNR2*, *DIRAS2*, *ESRP2*, *FAM83C*, *KCNT2*, *USH1G*		TCGA	103 primary and 368 metastatic melanomas	[74]
*AKR1C3*, *BMP1*, *CRTAC1*, *ECEL1*, *ERC2*, *FAM110C*, *FUT9*, *GABRA2*, *GAP43*, *GREM1*, *HECW1*, *KLHL1*, *KRT12*, *LHFPL4*, *NEFL*, *NEFM*, *NETO1*, *NKX2-2*, *NSG2*, *OCIAD2*, *OTOP1*, *PDE3B*, *PTPRN2*, *PTPRT*, *SIGLEC15*, *SLC13A5*, *SLC9A2*, *SLITRK6*, *SNAP91*, *STON2*, *TAC1*, *VAT1L*, *WNT5A*, *ALX1*, *BRD7*, *DTD1*, *GRSF1*, *HCN1*, *LTA4H*, *OXCT1*, *PATJ*, *PLXNC1*, *SSBP4*, *TELO2*, *TMEM177*	prediction of clinical response to ICB	GSE144946	genetically engineered mouse model	[77]
*JUN*, *AXL*	prediction of poor prognosis and response to immunotherapy	LMC	687 primary melanomas	[70]

FFPE: Formalin-Fixed Paraffin-Embedded; FF: Fresh Frozen; LMC: Leeds Melanoma Cohort; ICB: immune checkpoint blockade.

**Table 4:** Roles of miRNAs in recurrence signature. Log2 fold change and adjusted *p*-value refer to the comparison of recurrent against non-recurrent CM samples.

miRNA	log2 Fold Change	Adjusted *p* Value	Role in the Literature
mir-155	0.441282	0.046899	Associated with tumor prognosis. Its inhibition causes retarded glucose metabolism and thus, reduces in vivo tumor growth [78,79].
mir-205	−3.69183	1.03 × 10^−14^	Is a tumor suppressor miRNA in breast cancer which inhibits cell proliferation and anchorage independent growth as well as cell invasion [79].
mir-376b	1.057248	0.002396	Controls autophagy by directly regulating intracellular levels of two key autophagy proteins, ATG4C and BECN1 [80].
mir-1226	0.393576	0.010158	Regulates MUC1 and thus, dendritic cells resting which in turn play an important role in STS recurrence [81]. Targets expression of the mucin 1 oncoprotein and induces cell death [82].
mir-1306	0.254205	0.027816	Promotes apoptosis of granulosa cells (GCs) as well as attenuates the TGF-β/SMAD signaling pathway targeting and impairing TGFBR2 [83].
mir-3652	0.549545	0.002342	N/A
mir-3917	0.388593	0.020348	Has been recognized as biomarker and used for the construction of a stomach adenocarcinoma (STAD) prognostic signature [84].

**Table 5:** Roles of miRNAs in metastasis signature. Log2 fold change and adjusted *p*-value refer to the comparison of metastatic against primary CM samples.

miRNA	log2 Fold Change	Adjusted *p* Value	Role in the Literature
mir-186	0.290805	0.000389	Regulates TGFβ by suppressing SMAD6-7 in colorectal cancer and inhibits cell proliferation in melanoma [85,86].
mir-671	−0.24244	0.027927	miR-671-5p reduces NSCLC (squamous carcinoma) metastasis [87]. Its upregulation slows down proliferation and metastasis of A375 melanoma cells [88].
mir-760	0.503684	0.012509	It has been found downregulated in several cancers that can act both as tumor suppressor and as oncomir [89].
mir-944	−3.41097	8.62 × 10^−37^	Suppresses EMT in colorectal cancer [90]. It has been reported as downregulated in hepatocellular carcinoma (HCC) and suppresses the malignancy of HCC by deactivating PI3K [91]. Its overexpression is correlated with poor prognosis in cervical cancer [92].
mir-1976	0.444564	0.000327	It has been identified as tumor suppressor in NSCLC [93]. Its downregulation has been correlated with worse overall survival in triple-negative breast cancer (TNBC) from TCGA [94].
mir-3610	0.339103	0.049814	It has been associated with sumoylation, a molecular signature in head and neck cancer [95].
mir-3615	0.245396	0.036379	Its upregulation is correlated with high TNM stage and high proliferation in HCC [96].
mir-6842	0.450524	0.003272	N/A

**Table 6:** The signature miRNAs alone and combined with clinical data predict tumor recurrence and metastasis. Cross validation results and results in previously unseen to the algorithm samples are presented in terms of accuracy-ACC, specificity-SP, and sensitivity-SEN.

Metrics	Cross-Validation			Unseen Test Samples		
	ACC	SP	SEN	ACC	SP	SEN
recurrence signature	91.51%	92.65%	91.29%	73.85%	79.09%	88.78%
recurrence signature + clinical data	96.51%	97.13%	96.07%	85.38%	88.35%	92.86%
metastasis signature	97.39%	96.67%	98.38%	82.09%	82.40%	98.10%

## Data Availability

Not applicable.

## Supplementary Materials

The following supporting information can be downloaded at: www.mdpi.com/article/10.3390/ijms23031299/s1.
