# Supplementary material for: Revisiting miRNA Association with Melanoma Recurrence and Metastasis from a Machine Learning Point of View
Source: Int J Mol Sci. 2022 Jan 24;23(3):1299. doi: 10.3390/ijms23031299 (PMC8836065; doi:10.3390/ijms23031299)
Supplement: Supplementary file 1 [file ijms-23-01299-s001.zip › ijms-1568477-supplementary.pdf]

Supplementary Methods

Data collection: miRNA expression quantification data were downloaded from the GDC Data Portal of NIH [1]. The miRNA-Seq miRNA expression quantification corresponded to a total of 452 files for 448 cases. The respective biospecimen, clinical data and sample sheets were also downloaded from the GDC Data Portal. Tumor recurrence information was not available in these summarized files, so we downloaded the full clinical data from Firebrowse [2]. The field "patient.new_tumor_events.new_tumor_event_after_initial_treatment" was selected as the one indicating recurrence and used as desired output in the recurrence prediction problem. The field "sample type" was selected as the one indicating primary or metastatic tumor and used as desired output in the metastasis prediction problem.

Data preprocessing: Recurrence: Custom python scripts were created to process the single miRNA expression quantification files and merge them in one file with 452 samples (expression files) and 1881 features (miRNAs). Tumor recurrence was also mined from the clinical data and matched to the samples as labels with python scripts. Two samples of type Solid Tissue Normal and 13 samples with no reported information on tumor recurrence (one identical with a Solid Tissue Normal) were excluded from further analysis giving us 438 samples with available tumor recurrence information. Out of these, 328 experienced a new tumor event and 110 did not. Among the 1881 miRNAs, we excluded from further analysis miRNAs with zero counts in more than 70% of the samples, thus narrowing the number of miRNAs down to 880 miRNAs.

Metastasis: Out of the 452 samples, two samples of type Solid Tissue Normal were excluded from further analysis and one sample of type "additional metastatic" was considered metastatic. Of the remaining 450 samples, 353 were metastatic and 97 were primary tumors. Custom python scripts were created to process the single miRNA expression quantification files and merge them in one file with 450 samples (expression files) and 1881 features (miRNAs). Among the 1881 miRNAs, we excluded from further analysis miRNAs with zero counts in more than 70% of the samples, thus narrowing the number of miRNAs down to 543 miRNAs.

Differential expression analysis: Statistical analysis was performed on the raw read counts of the 880 and 543 miRNAs (for recurrence and metastasis, respectively) with custom R scripts with the DeSeq2 method and correction of p-values for multiple testing was performed using the Benjamini-Hochberg FDR adjustment method [3]. Setting the adjusted p value threshold to 0.05, we identified 203 (for recurrence) and 200 (for metastasis) statistically significant differentially expressed miRNAs. Among the miRNAs with adjusted p value < 0.05, those with log fold change >1.5 and <−1.5 are presented in supplementary Table 1.

Co-expression networks: Recurrence: One co-expression network based on the 880 miRNAs was created for the recurrent samples and one for the non-recurrent samples with the Spearman correlation method, with minimum threshold 0.55 and interval of trust 95% through InSyBio Bionets tool [4]. For the category tumor recurrence, the network has 326 nodes 1834 edges and for the category no tumor recurrence 353 nodes 1483 edges. The two networks were compared with the Pagerank method with 90% confidence interval to find significantly altered miRNAs between them. The 31 miRNAs which were found to be significantly altered in the network level are presented in supplementary Table 2. Merging the 31 miRNAs altered in the network with the 203 statistically significant differentially expressed miRNAs resulted in the 7 miRNAs of the recurrence signature.

Metastasis: One co-expression network based on the 543 miRNAs was created for the metastatic samples and one for the primary tumors samples with the Spearman correlation method, with minimum threshold 0.55 and interval of trust 95% through InSyBio Bionets tool [4]. For the metastatic category, the network has 307 nodes 1081 edges and for the primary tumors category 272 nodes 1033 edges. The two networks were compared with the Pagerank method with 90% confidence interval to find significantly altered miRNAs between them. The 24 miRNAs which were found to be significantly altered in the network level are presented in supplementary Table 3. Merging the 24 miRNAs altered in the network with the 200 statistically significant differentially expressed miRNAs resulted in the 8 miRNAs of the metastasis signature.

Classification models: The classification models for tumor recurrence and metastasis were generated with a hybrid ensemble heuristic optimization and classification method incorporated in the InSyBio Biomarkers tool [5]. The heuristic optimization algorithm is used to (a) identify the optimal feature subset to be used as input to the classifiers, to (b) select the most appropriate classifier among Support Vector Machines and Random Forests and (c) select the optimal parameters for the classifier, namely C and gamma of SVM and number of trees for Random Forests. A multi-objective Pareto based approach was used to reveal all the non-dominated solutions of the above-stated optimization problem [6]. The weights used for the goals were Selected Features Number Minimization 1, Accuracy 10, F1 score 10, F2 score 1, Precision 1, Recall 1, ROC_AUC 1, Number of SVs or Trees Minimization 1. Multiple models performing equally well on the user-defined goals are the final outcome. These models are then combined in an ensemble manner to predict tumor recurrence and metastasis [7].

The recurrence and the metastasis datasets were preprocessed with arithmetic sample-wise normalization and split in training sets (70% of the samples) and external test sets (30% of the samples) maintaining the proportion of recurrent vs. non-recurrent samples (1st classification problem) and metastatic vs. primary tumors samples (2nd classification problem). The two training sets were used to generate classifiers and 5-fold cross-validation was used to evaluate their predictive performance. For recurrence, the cross-validation accuracy achieved was 91.51% with 92.65% specificity and 91.29% sensitivity. For metastasis, the cross-validation accuracy achieved was 97.39% with 96.67% specificity and 98.38% sensitivity. Machine learning algorithms have the ability to learn linear and non-linear patterns from the data provided to them for their training. Thus, testing the performance of a classification models with the training data, or even with a cross-validation strategy favors the performance of the classifier. A common practice to more strictly evaluate a classifier is to calculate the predictive performance in samples not seen before by the algorithm. Thus, we subsequently evaluated our models on the external test sets (i.e., samples not seen before by the algorithm) achieving 73.85% accuracy with 79.09% specificity and 88.78% sensitivity in recurrence prediction. The respective metrics in the external test set for metastasis prediction were 88.78% accuracy with 82.40% specificity and 98.10% sensitivity.

Incorporation of clinical data: The clinical features explored for predicting tumor recurrence were sample_type, age_at_index, ethnicity, gender, race, ajcc_pathologic_m, ajcc_pathologic_n, ajcc_pathologic_stage, ajcc_pathologic_t, ajcc_staging_system_edition, primary_diagnosis, prior_malignancy, prior_treatment, patient_tumor_tissue_site (with the naming convention kept as in the original data). The feature ajcc_pathologic_stage had more than 10% missing values and was excluded from further analysis. The remaining features were preprocessed with arithmetic sample-wise normalization and k-nn missing values imputation. These clinical features were merged with the 7 miRNAs of the recurrence signature and together increased the predictive performance on the external

test set from 73.85% accuracy, 79.09% specificity and 88.78% sensitivity to 85.38% accuracy, 88.35% specificity and 92.86% sensitivity. The combined signature that yields these results consists of 5 miRNAs hsa-mir-1226, hsa-mir-1306, hsa-mir-205, hsa-mir-376b, hsa-mir-3917 and 3 clinical features Sample_Type, ajcc_pathologic_t and ajcc_staging_system_edition.

miRNA target genes: CM RNA-Seq data for the same patients' samples were downloaded from the GDC Data Portal of NIH. Genes with zero counts in more than 70% of the samples were excluded from further analysis. The Spearman correlation method was used to identify correlations of each of the signature miRNAs to genes. Genes negatively correlated to miRNAs are putative targets. Setting the Spearman rho threshold to <-0.25 reveals 947 genes negatively correlated to the recurrence signature miRNAs and 1419 genes negatively correlated to the metastasis signature miRNAs. The signature miRNAs were also searched for predicted targets in the miRDB database and ncRNASeq tool with confidence scores 0.65 and 0.3, respectively [8,9]. Merging the results, reveals 136 genes as potential targets of the recurrence signature miRNAs and 245 genes as potential targets of the metastasis signature miRNAs.

Functional and pathway enrichment analysis: Functional and pathway enrichment analysis was performed with DAVID v6.8 tool. The 136 miRNA target genes associated with recurrence and the 245 miRNA target genes associated with metastasis were explored for enrichment in GO biological processes, GO molecular functions and KEGG pathways. The terms they were enriched in with a p value threshold of 0.05 are presented in supplementary Tables 4 and 5, respectively.

Visualizations: Visualizations were performed with the R package corrplot and Cytoscape v3.8.2.

Supplementary References

[1] Gao, G. F., Parker, J. S., Reynolds, S. M., Silva, T. C., Wang, L. B., Zhou, W., ... & Noble, M. S. (2019). Before and after: comparison of legacy and harmonized TCGA genomic data commons' data. Cell systems, 9(1), 24-34.

[2] Broad Institute TCGA Genome Data Analysis Center. (2016). Analysis-ready standardized TCGA data from Broad GDAC Firehose 2016_01_28 run [Data set]. Broad Institute of MIT and Harvard. https://doi.org/10.7908/C11G0KM9

[3] Love, M. I., Huber, W., & Anders, S. (2014). Moderated estimation of fold change and dispersion for RNA-seq data with DESeq2. Genome biology, 15(12), 1-21.

[4] Theofilatos, K., Dimitrakopoulos, C., Alexakos, C., Korfiati, A., Likothanassis, S., & Mavroudi, S. (2016). InSyBio BioNets: an efficient tool for network-based biomarker discovery. EMBnet. journal, 22, 871.

[5] Theofilatos, K., Korfiati, A., Mavroudi, S., Cowperthwaite, M. C., & Shpak, M. (2019). Discovery of stroke-related blood biomarkers from gene expression network models. BMC medical genomics, 12(1), 1-15.

[6] Corthésy, J., Theofilatos, K., Mavroudi, S., Macron, C., Cominetti, O., Remlawi, M., ... & Dayon, L. (2018). An adaptive pipeline to maximize isobaric tagging data in large-scale MS-based proteomics. Journal of proteome research, 17(6), 2165-2173.

[7] Rapakoulia, T., Theofilatos, K., Kleftogiannis, D., Likothanasis, S., Tsakalidis, A., & Mavroudi, S. (2014). EnsembleGASVR: a novel ensemble method for classifying missense single nucleotide polymorphisms. Bioinformatics, 30(16), 2324-2333.

[8] Chen, Y., & Wang, X. (2020). miRDB: an online database for prediction of functional microRNA targets. Nucleic acids research, 48(D1), D127-D131.

[9] Korfiati, A., Theofilatos, K., Alexakos, C., & Mavroudi, S. (2017). InSyBio ncRNASeq: a web tool for analyzing non-coding RNAs. EMBnet. journal, 23, 882.

Supplementary Table S1. Differentially expressed miRNAs in recurrence and metastasis with adjusted p value <0.05. Only miRNAs with log fold change >1.5 and <-1.5 are presented.

miRNA	Recurrence log2 Fold Change	Metastasis log2 Fold Change
hsa-mir-519d	4.10	-
hsa-mir-206	2.92	-
hsa-mir-519a-2	2.88	-
hsa-mir-520b	2.49	-
hsa-mir-133b	2.31	-
hsa-mir-519a-1	2.27	1.85
hsa-mir-526b	2.24	1.94
hsa-mir-520a	2.22	-
hsa-mir-1323	2.13	-
hsa-mir-675	1.84	1.92
hsa-mir-512-1	1.78	-
hsa-mir-518a-2	1.77	-
hsa-mir-518b	1.74	-
hsa-mir-512-2	1.72	-
hsa-mir-135a-1	1.61	-
hsa-mir-137	1.60	-
hsa-mir-3923	1.52	-
hsa-mir-153-2	1.21	1.55
hsa-mir-203a	-3.71	-5.35
hsa-mir-205	-3.69	-5.56
hsa-mir-203b	-3.03	-

hsa-mir-944	-2.51	-3.41
hsa-mir-200c	-2.00	-2.99
hsa-mir-141	-1.89	-2.68
hsa-mir-892a	-1.63	-
hsa-mir-891a	-1.59	-1.94
hsa-mir-200b	-1.58	-2.88
hsa-mir-200a	-1.47	-2.42
hsa-mir-224	-1.16	-1.58

Supplementary Table S2. 31 miRNAs significantly altered between the co-expression network of recurrent samples and the co-expression network of non-recurrent samples. Their log2 fold change and adjusted p value are also presented.

miRNA	log2 Fold Change	Adjusted p value
hsa-mir-1180	-0.02	0.92
hsa-mir-1226	0.39	0.01
hsa-mir-1291	-0.12	0.65
hsa-mir-1301	0.03	0.84
hsa-mir-1306	0.25	0.03
hsa-mir-155	0.44	0.05
hsa-mir-193a	0.09	0.60
hsa-mir-205	-3.69	1.03E-14
hsa-mir-25	0.02	0.87
hsa-mir-339	-0.12	0.41
hsa-mir-345	0.04	0.83
hsa-mir-3614	0.02	0.95
hsa-mir-3652	0.55	2.34E-03
hsa-mir-3680-1	0.21	0.31

hsa-mir-3680-2	0.11	0.70
hsa-mir-376b	1.06	2.40E-03
hsa-mir-3917	0.39	0.02
hsa-mir-423	-0.02	0.81
hsa-mir-4533	-0.51	0.36
hsa-mir-4725	0.08	0.84
hsa-mir-4772	0.38	0.08
hsa-mir-516b-2	1.48	0.06
hsa-mir-548ag-2	0.15	0.81
hsa-mir-5699	0.05	0.81
hsa-mir-589	0.03	0.85
hsa-mir-616	0.26	0.14
hsa-mir-652	0.08	0.59
hsa-mir-671	-0.19	0.10
hsa-mir-760	0.21	0.37
hsa-mir-877	-0.15	0.40
hsa-mir-93	-0.20	0.16

Supplementary Table S3. 24 miRNAs significantly altered between the co-expression network of metastatic samples and the co-expression network of primary tumors samples. Their log2 fold change and adjusted p value are also presented.

miRNA	log2 Fold Change	Adjusted p value
hsa-mir-324	-0.02717	0.822165
hsa-mir-345	0.200647	0.133668
hsa-mir-328	-0.12088	0.421828
hsa-mir-616	0.165563	0.344759

hsa-mir-3610	0.339103	0.049814
hsa-mir-3605	0.230905	0.069731
hsa-mir-589	0.041708	0.746174
hsa-mir-1248	0.194373	0.241891
hsa-mir-671	-0.24244	0.027927
hsa-mir-339	-0.02803	0.830344
hsa-mir-185	-0.09129	0.466247
hsa-mir-760	0.503684	0.012509
hsa-mir-186	0.290805	0.000389
hsa-mir-4772	0.043223	0.844644
hsa-mir-30c-1	-0.16449	0.225337
hsa-mir-3615	0.245396	0.036379
hsa-mir-6842	0.450524	0.003272
hsa-mir-6125	0.317634	0.126056
hsa-mir-3680-2	0.302132	0.180251
hsa-mir-25	-0.02416	0.822165
hsa-mir-210	0.29431	0.136062
hsa-mir-1976	0.444564	0.000327
hsa-mir-4443	0.347378	0.225337
hsa-mir-944	-3.41097	8.62E-37

Supplementary Table S4. Functional enrichment analysis of the 136 genes targets of the 7 recurrence miRNAs

Category	Term	P-Value	Fold Enrichment
BP	transcription, DNA-templated	4.60E-06	2.342525
MF	metal ion binding	3.00E-05	2.175737

MF	protein binding	6.10E-05	1.3451
MF	ATP binding	2.80E-04	2.258328
MF	transcription factor activity, sequence-specific DNA binding	1.20E-03	2.488528
MF	siRNA binding	1.30E-03	52.75313
MF	nucleic acid binding	1.50E-03	2.427893
BP	protein phosphorylation	1.60E-03	3.347687
MF	protein tyrosine kinase activator activity	1.70E-03	46.89167
MF	chromatin binding	1.80E-03	3.597826
MF	DNA binding	3.00E-03	1.932811
BP	negative regulation of transcription, DNA-templated	3.10E-03	3.059209
BP	regulation of transcription, DNA-templated	4.80E-03	1.937709
MF	protein kinase C activity	4.90E-03	28.135
BP	regulation of autophagy	5.50E-03	11.10215
BP	phosphorylation	5.70E-03	6.938843
BP	phosphatidylinositol-mediated signaling	7.00E-03	6.546078
MF	kinase activity	7.20E-03	4.085996
MF	proline-rich region binding	7.80E-03	22.21184
MF	methyl-CpG binding	9.50E-03	20.09643
BP	positive regulation of transcription from RNA polymerase II promoter	1.00E-02	2.12197
BP	embryonic pattern specification	1.20E-02	18.10133

KEGG_PATHWAY	Proteoglycans in cancer	1.20E-02	4.211633
BP	peptidyl-serine phosphorylation	1.20E-02	5.551074
KEGG_PATHWAY	Adherens junction	1.30E-02	7.909169
MF	protein kinase activity	1.40E-02	3.134819
BP	convergent extension	1.40E-02	138.7769
BP	neural crest formation	1.40E-02	138.7769
BP	positive regulation of protein tyrosine kinase activity	1.50E-02	16.01271
MF	signal transducer activity	1.50E-02	4.1375
KEGG_PATHWAY	Wnt signaling pathway	1.50E-02	5.086513
BP	macroautophagy	1.70E-02	7.304045
MF	protein kinase binding	1.70E-02	2.993085
BP	insulin receptor signaling pathway	1.80E-02	7.116762
BP	regulation of phosphatidylinositol 3-kinase signaling	1.80E-02	7.116762
KEGG_PATHWAY	Aldosterone synthesis and secretion	1.90E-02	6.932729
BP	face morphogenesis	1.90E-02	13.87769
KEGG_PATHWAY	Ras signaling pathway	2.00E-02	3.727109
KEGG_PATHWAY	Fc gamma R-mediated phagocytosis	2.00E-02	6.685131
BP	RIG-I signaling pathway	2.10E-02	92.51791
KEGG_PATHWAY	ErbB signaling pathway	2.20E-02	6.454609
MF	beta-tubulin binding	2.70E-02	11.72292

KEGG_PATHWAY	Cysteine and methionine metabolism	2.90E-02	11.08324
KEGG_PATHWAY	Melanogenesis	3.20E-02	5.61551
BP	protein tetramerization	3.30E-02	10.40826
BP	dsRNA transport	3.50E-02	55.51074
BP	signal transduction by protein phosphorylation	3.90E-02	9.462059
BP	covalent chromatin modification	4.70E-02	4.912455
KEGG_PATHWAY	MicroRNAs in cancer	4.80E-02	2.945198
BP	extracellular matrix assembly	4.90E-02	39.65053
MF	protein serine/threonine kinase activity	5.00E-02	2.618949

Supplementary Table S5. Functional enrichment analysis of the 245 genes targets of the 8 metastasis miRNAs

Category	Term	PValue	Fold Enrichment
BP	regulation of transcription, DNA-templated	9.82E-10	2.56
BP	transcription, DNA-templated	2.48E-07	2.09
BP	negative regulation of transcription from RNA polymerase II promoter	6.95E-06	2.78
KEGG_PATHWAY	AMPK signaling pathway	1.00E-05	6.99
MF	transcription factor binding	3.48E-04	3.51
KEGG_PATHWAY	Prostate cancer	4.86E-04	6.84
BP	anterior/posterior pattern specification	5.89E-04	6.74
MF	chromatin binding	6.07E-04	2.94
MF	protein binding	1.02E-03	1.21
MF	metal ion binding	1.13E-03	1.63
MF	sequence-specific DNA binding	1.22E-03	2.52
MF	zinc ion binding	1.23E-03	1.90
BP	gastrulation with mouth forming second	1.27E-03	18.12
BP	dendrite morphogenesis	1.28E-03	10.41
MF	mitogen-activated protein kinase binding	1.52E-03	17.05
MF	1-phosphatidylinositol-3-phosphate 4-kinase activity	1.63E-03	46.04

KEGG_PATHWAY	cGMP-PKG signaling pathway	2.14E-03	4.35
MF	core promoter sequence-specific DNA binding	2.29E-03	8.92
KEGG_PATHWAY	Fc gamma R-mediated phagocytosis	2.67E-03	6.14
MF	DNA binding	3.13E-03	1.65
BP	cellular response to insulin stimulus	3.20E-03	6.00
KEGG_PATHWAY	Axon guidance	3.25E-03	4.74
MF	1-phosphatidylinositol-4-phosphate 5-kinase activity	3.37E-03	32.89
MF	14-3-3 protein binding	3.56E-03	12.79
MF	mRNA 3'-UTR binding	3.70E-03	7.83
KEGG_PATHWAY	FoxO signaling pathway	4.24E-03	4.49
KEGG_PATHWAY	HIF-1 signaling pathway	4.76E-03	5.37
BP	cellular response to hydrogen peroxide	6.28E-03	6.76
KEGG_PATHWAY	cAMP signaling pathway	7.41E-03	3.47
KEGG_PATHWAY	Insulin resistance	7.80E-03	4.78
MF	nucleic acid binding	9.18E-03	1.79
KEGG_PATHWAY	Chronic myeloid leukemia	9.19E-03	5.97
KEGG_PATHWAY	Thyroid hormone signaling pathway	1.01E-02	4.49
MF	ATP binding	1.09E-02	1.59
KEGG_PATHWAY	Sphingolipid signaling pathway	1.20E-02	4.30
BP	hair follicle development	1.29E-02	8.11
BP	retrograde transport, endosome to plasma membrane	1.37E-02	16.51
MF	histone deacetylase activity	1.40E-02	7.87
BP	regulation of mitotic cell cycle	1.48E-02	7.70
BP	signal transduction	1.53E-02	1.66
BP	positive regulation of insulin secretion	1.58E-02	7.51
BP	positive regulation of transcription from RNA polymerase II promoter	1.61E-02	1.73
BP	axon guidance	1.73E-02	3.39
KEGG_PATHWAY	Type II diabetes mellitus	1.74E-02	7.17
KEGG_PATHWAY	Progesterone-mediated oocyte maturation	1.75E-02	4.94
MF	RNA polymerase II core promoter proximal region sequence-specific DNA binding	1.77E-02	2.38
BP	multicellular organism development	1.82E-02	2.07
KEGG_PATHWAY	Cocaine addiction	1.84E-02	7.02
BP	regulation of phosphatidylinositol 3-kinase signaling	1.84E-02	4.94
BP	insulin receptor signaling pathway	1.84E-02	4.94
BP	histone deacetylation	2.15E-02	6.70
BP	neuron projection morphogenesis	2.40E-02	6.42
MF	phosphatidylinositol 3-kinase binding	2.48E-02	12.12

KEGG_PATHWAY	Hepatitis B	2.52E-02	3.56
MF	protein serine/threonine kinase activity	2.53E-02	2.24
KEGG_PATHWAY	Phosphatidylinositol signaling system	2.58E-02	4.39
KEGG_PATHWAY	Regulation of lipolysis in adipocytes	2.61E-02	6.14
KEGG_PATHWAY	Estrogen signaling pathway	2.67E-02	4.34
BP	regulation of autophagy	2.67E-02	6.16
KEGG_PATHWAY	Choline metabolism in cancer	2.84E-02	4.26
BP	spermatogenesis	2.85E-02	2.20
KEGG_PATHWAY	mTOR signaling pathway	2.86E-02	5.93
MF	GTPase activator activity	2.95E-02	2.48
BP	rhythmic process	3.26E-02	5.71
BP	phosphatidylinositol phosphorylation	3.36E-02	4.10
KEGG_PATHWAY	Apoptosis	3.40E-02	5.55
KEGG_PATHWAY	TNF signaling pathway	3.42E-02	4.02
BP	neuron differentiation	3.47E-02	4.05
BP	positive regulation of collagen biosynthetic process	3.52E-02	10.05
KEGG_PATHWAY	Pathways in cancer	3.53E-02	2.19
BP	axon extension	3.81E-02	9.63
BP	extracellular matrix constituent secretion	3.83E-02	51.35
KEGG_PATHWAY	Glioma	3.83E-02	5.29
KEGG_PATHWAY	Cholinergic synapse	3.83E-02	3.87
MF	phosphatidylinositol-3,4-bisphosphate 5-kinase activity	3.84E-02	51.15
BP	phosphatidylinositol biosynthetic process	3.91E-02	5.31
KEGG_PATHWAY	Transcriptional misregulation in cancer	4.25E-02	3.09
MF	transcription factor activity, sequence-specific DNA binding	4.46E-02	1.60
KEGG_PATHWAY	Ras signaling pathway	4.48E-02	2.66
KEGG_PATHWAY	Adipocytokine signaling pathway	4.61E-02	4.91
BP	hippo signaling	4.72E-02	8.56
BP	transcription initiation from RNA polymerase II promoter	4.75E-02	3.04
KEGG_PATHWAY	Prolactin signaling pathway	4.77E-02	4.84
KEGG_PATHWAY	Inositol phosphate metabolism	4.77E-02	4.84
KEGG_PATHWAY	Melanoma	4.77E-02	4.84
MF	transcription corepressor activity	4.93E-02	2.65
